# Electrophysiological, biomechanical, and finite element analysis study of sacral nerve injury caused by sacral fracture

**DOI:** 10.3389/fbioe.2022.920991

**Published:** 2022-09-21

**Authors:** Zisheng Xu, Yifei Jiang, Weidong Mu, Wenlong Li, Guanjun Zhang, Shichao Jiang, Peng Xu

**Affiliations:** ^1^ Department of Orthopedic Trauma, Shandong Provincial Hospital Affiliated to Shandong University, Jinan, China; ^2^ Department of Orthopaedic trauma, Shandong Provincial Hospital affiliated to Shandong First Medical University, Jinan, China; ^3^ Laiwu People’s Hospital, Jinan, China; ^4^ State Key Laboratory of Advanced Design and Manufacturing for Vehicle Body, Hunan University, Changsha, China

**Keywords:** constrictive injury, electrophysiology, biomechanics, finite element analysis, compression, lumbosacral trunk, nerve root, sacrum fracture

## Abstract

**Background:** We aimed to study the mechanism of sacral nerve injury caused by sacral fractures and the relationship between nerve decompression and nerve function.

**Methods:** First, we observed the anatomical features of lumbosacral nerve root region in Sprague-Dawley rats. Next, the rats were divided into the sham, 10 g, 30 g, and 60 g groups for electrophysiological studies on nerve root constriction injury. Then we studied the biomechanical properties of rat nerve roots, lumbosacral trunk, and sacrum. Finally, we established a finite element analysis model of sacral nerve roots injury in rats and determined the correlation between sacral deformation and the degree of sacral nerve roots injury.

**Result:** Anatomical study showed L5 constitutes sciatic nerve, the length of the L5 nerve root is 3.67 ± 0.15 mm, which is suitable for electrophysiological research on nerve root compression injury. After a series of electrophysiological study of L5 nerve roots, our results showed that nerve root function was almost unaffected at a low degree of compression (10 g). Nerve root function loss began at 30 g compression, and was severe at 60 g compression. The degree of neurological loss was therefore positively correlated with the degree of compression. Combining biomechanical testing of the lumbosacral nerve roots, finite element analysis and neuroelectrophysiological research, we concluded when the sacral foramina deformation is >22.94%, the sacral nerves lose function. When the compression exceeds 33.16%, early recovery of nerve function is difficult even after decompression.

**Conclusion:** In this study, we found that the neurological loss was positively correlated with the degree of compression. After early decompression, nerve root function recovery is possible after moderate compression; however, in severe compression group, the nerve function would not recover. Furthermore, FEA was used to simulate nerve compression during sacral fracture, as well as calculate force loading on nerve with different deformation rates. The relationship between sacral fractures and neurological loss can be analyzed in combination with neurophysiological test results.

## 1 Introduction

Sacral fracture is a serious type of pelvic fracture, and sacral nerve injury is often missed due to combined trauma in other parts. The incidence of sacral fractures with nervous system damage is about 60%, which is much higher than that of nervous system damage in pelvic fracture (Mehta et al., 2006). Disability in patients after sacral fracture involves a series of dysfunctions due to sacral nerve injury (Gansslen et al., 2003; Nicodemo et al., 2008). There are three types of injury: constriction, strain, and laceration injury, of which constriction injury is the most common. The majority of sacral fractures are Denis Zone Ⅰ and Ⅱ, and often cause nerve injury. The sacrum is easily fractured on exposure to high-energy trauma, resulting in sacral foramen deformation and shrinkage. The sacral fracture fragment compresses and stretches the sacral nerve, and can cause sacral nerve damage. The mechanism underlying sacral nerve constrictive injury is currently unknown, so there is no consensus on the optimal clinical treatment of sacral fractures combined with sacral nerve injury, including administering surgical decompression, choice of decompression methods, and the effectiveness of decompression (Taguchi et al., 1999; König et al., 2012)

Schildhauer et al. studied the imaging data of sacral fractures combined with nerve damage and found that when the sacral foramen compression area is 50%, surgical decompression should be performed, and when the sacral foramen compression area exceeds 75%, even if the operation is performed as soon as possible, nerve function is difficult to restore (Schildhauer et al., 2006). Therefore, the relationship between the degree of sacral foramen constriction and sacral nerve damage is of great significance in determining the timing of surgical decompression.

There have been few studies on the mechanism of sacral nerve constrictive injury caused by sacral fracture. The correlation between fracture compression and nerve injury remains unclear. Direct research on human sacral nerve damage is difficult to achieve (Geuna, 2015). The establishment of animal models and related anatomical studies is therefore important for studying the mechanism of sacral nerve constrictive injury.

The complex anatomy around the sacrum includes important blood vessels and nerves. Surgery is difficult and animal mortality is high, and fractures cause large nerve interference factors, making it impossible to avoid nerve damage during surgery.

In this study, we aimed to establish a reliable animal model to mimic sacral nerve constrictive injury to simulate the pathological process of different degrees of acute compression. Furthermore, we studied nerve function and the injury mechanism by using neuroelectrophysiological methods. In addition, we aimed to simulate nerve decompression and observe the changes in nerve function after different compression durations to provide a theoretical basis for the clinical treatment of clinical sacral nerve injury. Finite element analysis obtains virtual specimens by establishing a three-dimensional living body structure model, wherein we can study the relationship between sacral fracture and nerve injury by loading different experimental conditions. In previous study, we studied pelvic displacement and lumbosacral trunk nerve traction injury, and built a material testing platform for nerve traction injury *in vivo*. Unlike lumbosacral trunk injury, the sacral nerve is mainly injured by compression. Neuroelectrophysiology is therefore useful to obtain the nerve injury threshold to define material failure, and the sacral nerve material parameters are calculated in combination with biomechanical tests. Sacral-sacral nerve geometric data was then used to establish a finite element model to visually study the mechanism of sacral nerve injury, and assign biomechanical material properties measured by the material mechanics method to the model, establish a virtual “experimental sample”, and simulate the model under experimental conditions (Wheeldon et al., 2006) such as stiffness, structural deformation of any part, stress/strain distribution, internal energy change, fuzzy limit, and other changes. With this modeling method, it is difficult to establish an effective and accurate model to obtain accurate parameters. The best option is to measure nerve material parameters through experiments, and use computer simulation.

## 2 Materials and methods

### 2.1 Anatomy and electrophysiological study of lumbosacral nerve roots in SD rats

Twenty healthy SD adult rats (9 weeks old, weight: 250–300 g, male and female in a random proportion) provided by the Experimental Animal Center of Shandong Provincial Hospital were used for anatomy research. For the electrophysiological tests, rats were divided into the sham (sham operation group), 10 g, 30 and 60 g groups, each with 24 rats, and tested on days 0, 2, 7, and 14 (Table 1). All animal experiment protocols met national standards and were approved by the ethics committee of the Provincial Hospital Affiliated to Shandong University.

**TABLE 1:** Experimental groups: sham, 10, 30, 60 g, 6 animals in each group. Test at 0, 2, 7, and 14 days respectively.

Group (d)	Sham	10 g	30 g	60 g
0	6	6	6	6
2	6	6	6	6
7	6	6	6	6
14	6	6	6	6

### 2.2 Anatomy of lumbosacral nerve roots in experimental animals

Adult SD rats (weight: 250–300 g) were anesthetized with 2% pentobarbital (50 mg/kg, intraperitoneal injection) and placed in the prone position. The L4-S1 nerve roots were surgically exposed, and the nerve root anatomy observed under an operating microscope (×10). The diameter of the intervertebral foramen, the length and diameter of the nerve root in the spinal canal, and the diameter of the spinal nerve formed after exiting the intervertebral foramen were measured using a vernier caliper (with an accuracy of 0.02 mm). The animal was then fixed in a supine position, and a midline abdominal incision was made. In order to better measure the anatomical structure of the sacral foramen, the sacrum, and the anatomical parameters, the pelvic contents were removed and the bilateral pubic and ischial branches were clipped off. The lumbosacral plexus was dissected and the composition of the femoral, sciatic, and pudendal nerves were observed under an operating microscope (×10).

### 2.3 Establishment of a rat model of nerve root compression injury

SD adult rats (weight: 250–300 g) were anesthetized with 2% pentobarbital (50 mg/kg, intraperitoneal injection), and a cut of about 3 cm was made in the back skin with tissue scissors. The paravertebral muscles were separated, with attention to hemostasis, and the lamina was exposed and resected. The dura mater was cut to reveal the L5 nerve roots.

The operation was performed in all the rats by the same experimenter. L5 nerve roots were clamped for 10 s 3 times with sterile vascular clamps of 10 g, 30 g, or 60 g. The interval between the three clamps was 10 s. In the sham group, no clamping was performed. Electrophysiological tests were performed on days 0, 2, 7, and 14 after the rat nerve root injury model was constructed.

### 2.4 Electrophysiological detection of rat nerve root injury model

The rat model was anesthetized with 2% pentobarbital, and the posterior root of the L5 nerve was exposed. After connecting the bioelectrophysiological system to the computer and the electrodes, the two recording electrodes (R1, R2) were placed on the proximal 10 mm of the L5 posterior nerve root. A transverse incision was made at the middle and lower 1/3 level of the line connecting the superior posterior iliac spine and the ischial tuberosity. The skin and subcutaneous tissue were cut to expose the gluteus maximus, which was bluntly separated to expose the piriformis and the sciatic nerve. Stimulation electrodes were placed on the sciatic nerve, and 1, 2, and 3 V voltages were used for electrical stimulation. Electrical signals recorded by the two recording electrodes (R1, R2) were analyzed using the Clampex system. Analysis data included nerve conduction velocity (conduction velocity, CV), compound action potential under the curve area (AUC), and nerve compound action potential peak value. The von-Frey pain test was then performed as follows: the innervated area of the sciatic nerve of the rat foot was stimulated with von-Frey hair, and the force that caused the nerve to discharge was recorded.

### 2.5 Electrophysiological testing indicators

In Denis Zone Ⅱor Ⅲ type fracture, the displaced fracture fragments compress nerve and cause loss of function. Electrophysiological methods can show changes in nerve action potentials. The main indicators include: CV, AUC, and nerve compound action potential peak value. Of these, nerve conduction velocity represents nerve conduction function, and the area under the curve of compound action potential (AUC) and peak value represent functional changes after nerve activation.

### 2.6 Biomechanical study of the nerve root, lumbosacral trunk and sacrum

#### 2.6.1 Biomechanics of rat nerve root and lumbosacral trunk

##### 2.6.1.1 Removal of rat nerve roots and lumbosacral trunk

Adult SD male rats (250–300 g) were euthanized by overdose with pentobarbital (50 mg/ml). The lamina, articular process, and transverse process were exposed and the posterior lamina was extensively cut to the intervertebral foramen under a microscope. After the dura mater was cut, the posterior root of the L5 nerve was carefully exposed and dissected, and was cut and removed with fiber scissors before the test. Mechanical tests were performed with freshly collected samples.

##### 2.6.1.2 Tension test of rat nerve root and lumbosacral trunk

**FIGURE 1:**
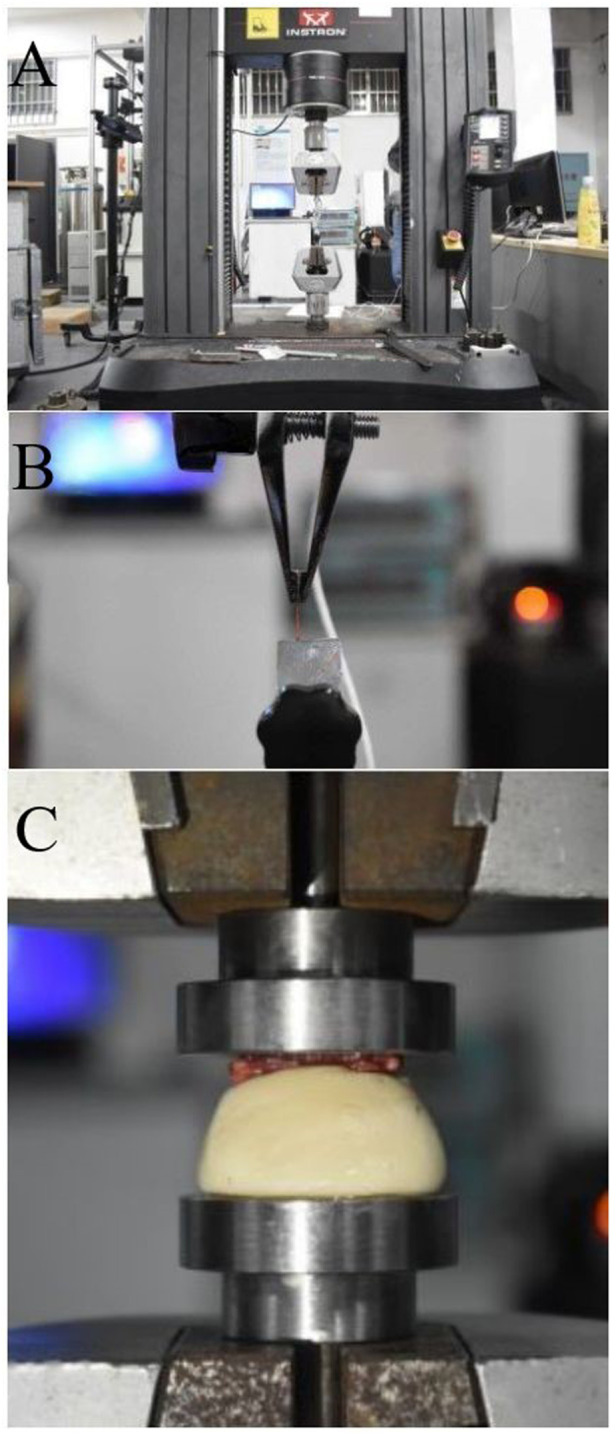
**(A)** Image of the INSTRON 5985 machine, **(B)** Nerve axial tensile test, **(C)** Biomechanical testing of the rat sacrum: bone compression test.

Before stretching, the original diameter of each L5 nerve root and lumbosacral trunk was measured using a vernier caliper. The INSTRON 5985 (Instron Corp, Norwood, MA, United States) testing machine (Figure 1A) was used to uniformly test tensile strength of the rat nerve roots. The stretching speed was set to 0.01 mm/s (Figure 1B), and a preload of 0.02 N was applied to the sample before the experiment. Data such as nerve displacement was converted into electrical signals in the function recorder. When the nerve root and lumbosacral trunk were stretched to destruction, the diameter was measured again within 1 min after the test to obtain load and displacement data. Maximum stress and strain, ultimate load, elastic variables, and material parameters were determined based on the load-displacement measurement results. Based on the average diameter of the sacral nerve and lumbosacral trunk, the tensile load is divided by the constant nerve cross-sectional area to obtain the stress. The original length of the shift quotient or elongation of the test nerve were used to calculate the strain. The elastic variable (a measure of material stiffness) was calculated based on the slope of the rising part of the stress-strain curve. These data were combined to compare the mechanical properties of the two nerve materials and obtain the sacral nerve material parameters. The soft tissue was divided into lumbosacral trunk and nerve roots, and 11 samples of lumbosacral trunk and 12 samples of nerve roots were tested.

#### 2.6.2 Biomechanical testing of rat sacrum

Six SD male rats (weight: 250–300 g) were euthanized with an overdose of pentobarbital (50 mg/ml). The INSTRON 5985 testing machine was used to perform a uniform compression test on the rat sacrum, and the compression duration was standardized to 30 s to ensure stable compression of the sacrum. The sacrum was compressed at a speed of 0.02 mm/s until it was completely deformed (Figure 1C). A 0.2 N preload was applied to the sample before the experiment, and the downward vertical pressure was applied through the upper clamp. Data such as the compression degree of the sacrum fracture and the pressure received were converted into electrical signals in the function recorder. The transverse compression force and displacement change of the sacrum during the experiment and the force when the sacrum was crushed were recorded, and the compression force-displacement curve was obtained.

### 2.7 Finite element analysis of sacral nerve constriction injury caused by sacral fracture

Finite element analysis was performed using the LS-Dyna finite element simulation solution software. We used ICEM and Hypermesh software for image processing.

#### 2.7.1 Neural material parameter calculation

**FIGURE 2:**
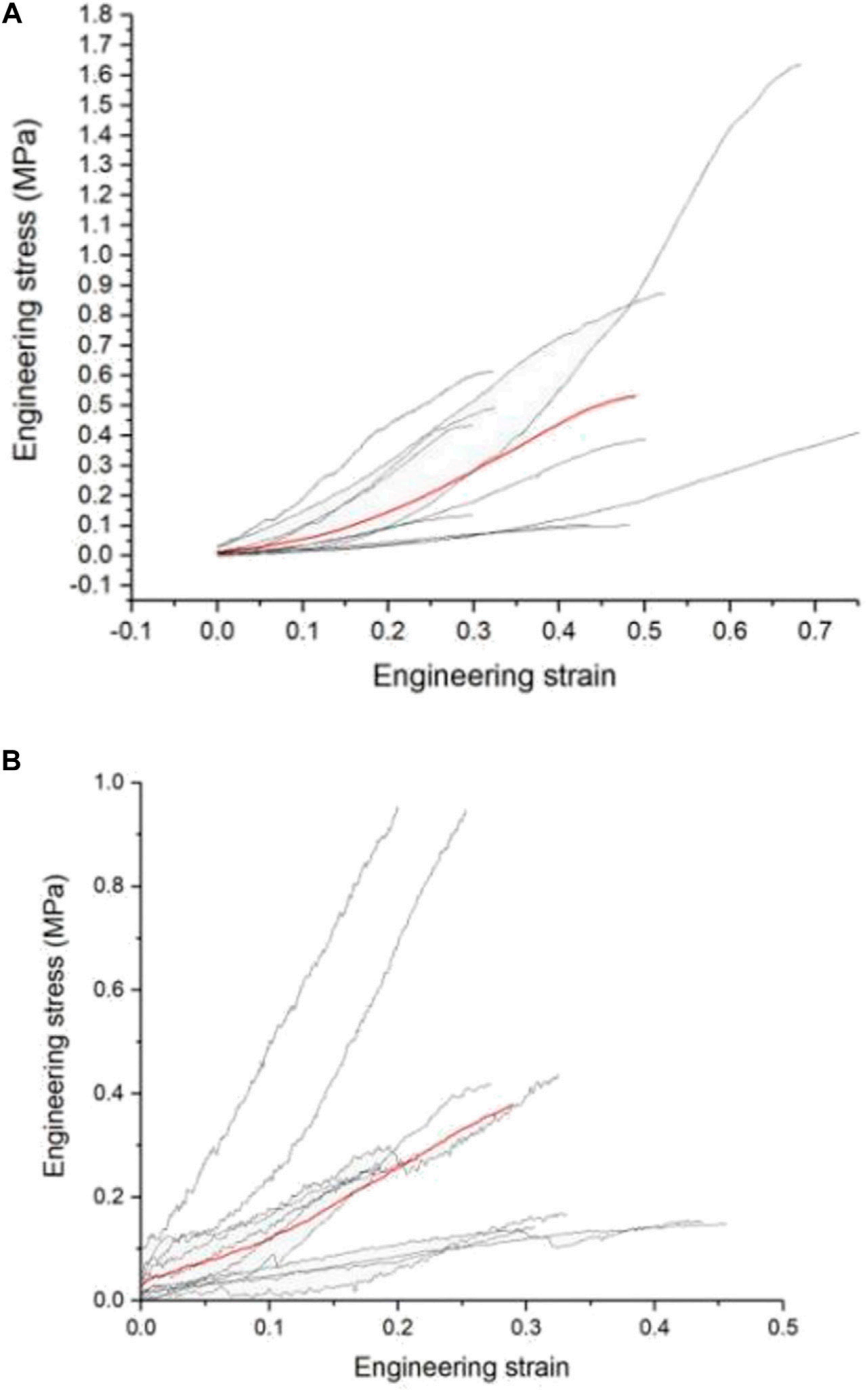
**(A)** Engineering stress-strain curve of the lumbosacral trunk, **(B)** Engineering stress-strain curve of the nerve root.

This study uses images to obtain the geometric parameters of the lumbosacral trunks and nerve roots before the tensile test, and assumes that the nerve is a homogeneous cylinder. The geometric parameters of each sample (Table 2) are shown. According to the classical mechanics formula of material tensile test, the engineering stress-strain curve of each sample can be obtained, which is the biomechanical properties of nerve materials not depending on geometry, as shown in Figure 2A and Figure 2B. Among them, the red curve represents the average engineering stress-strain curve. In Figure 2A, the average ultimate stress of the lumbosacral trunk is 0.530 MPa, and the average ultimate strain is 48.9%. In Figure 2B, the average ultimate stress of the nerve root is 0.377 MPa, and the average ultimate strain is 28.9%. Therefore, it can be predicted that the maximum stress that the lumbosacral trunk can withstand under tensile load is 0.530 MPa, while the nerve root is 0.377 MPa.

**TABLE 2:** Geometric parameters of nerve specimen.

Serial number	Sample type	Sample size before experiment (mm)	Diameter	Tensile cross-sectional area
002_NR	Nerve Root	8	0.5	0.196
003_NR	Nerve Root	6.5	0.6	0.283
005_NR	Nerve Root	5	0.6	0.283
006_NR	Nerve Root	11	0.6	0.283
007_LS	Lumbosacral Trunk	7	1	0.785
008_LS	Lumbosacral Trunk	7	1	0.785
009_NR	Nerve Root	8	0.5	0.196
010_NR	Nerve Root	9	0.5	0.196
011_LS	Lumbosacral Trunk	8	1.1	0.95
012_LS	Lumbosacral Trunk	5	1.2	1.13
013_LS	Lumbosacral Trunk	4	1.6	2.01
014_LS	Lumbosacral Trunk	3	0.8	0.502
015_NR	Nerve Root	6	0.7	0.385
016_NR	Nerve Root	9	0.4	0.126
017_LS	Lumbosacral Trunk	4	1.3	1.327
018_LS	Lumbosacral Trunk	3	1.7	2.269
019_NR	Nerve Root	3	1	0.785
020_NR	Nerve Root	6	0.5	0.196
022_LS	Lumbosacral Trunk	4	0.9	0.636
024_LS	Lumbosacral Trunk	3	0.9	0.636
025_NR	Nerve Root	6	0.7	0.385
026_NR	Nerve Root	10	0.6	0.283
028_LS	Lumbosacral Trunk	3	0.6	0.283

#### 2.7.2 Simulation analysis of nerve injury during sacral fracture

In order to simulate the stress state of the sacral nerve during a sacral fracture, it is first necessary to estimate the parameters of nerve materials. In this study, the material parameters of the nerve were determined by simulating the tensile test. The material parameters were then used to simulate the load that the nerve could bear when compressed.

#### 2.7.3 Nerve root axial tensile test simulation

**FIGURE 3:**
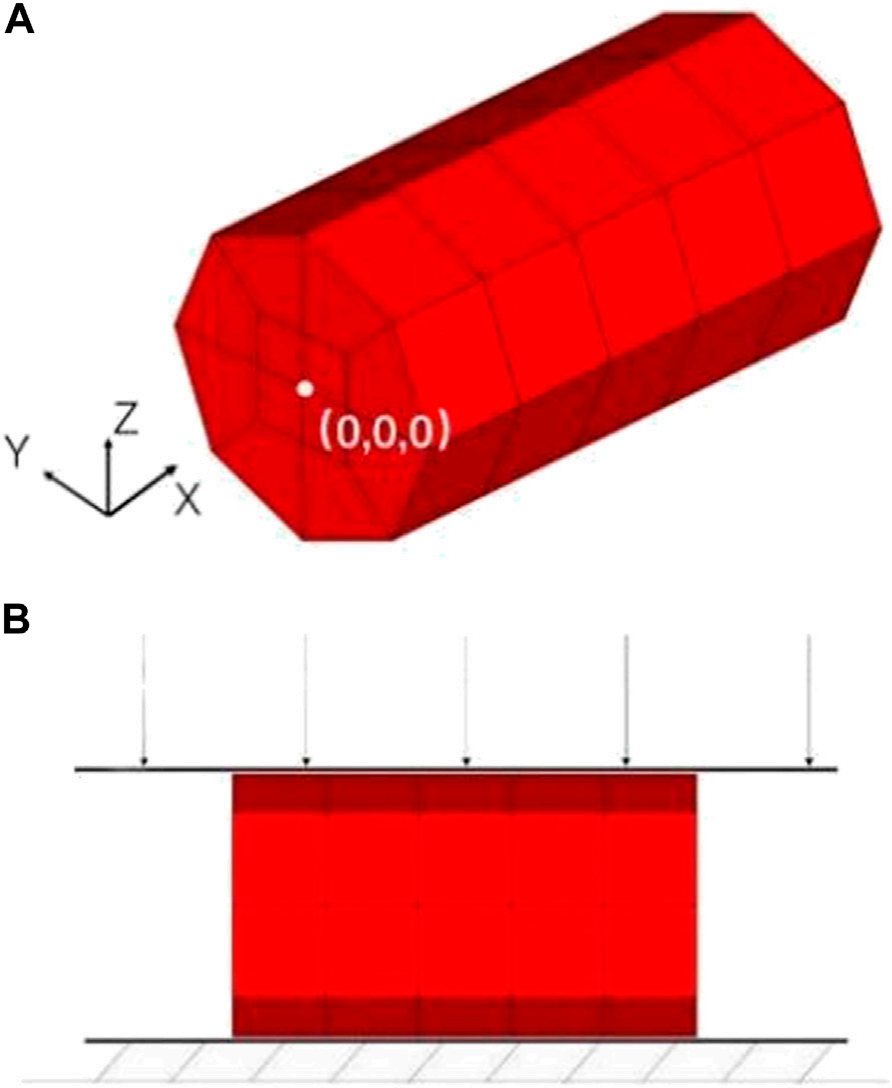
**(A)** Finite element model of the nerve root, **(B)** Schematic diagram of nerve root radial compression test simulation.

The geometric model of the nerve root was simplified into a cylindrical structure with a diameter of 1.7 mm and a height of 3 mm by using the reverse modeling method, and the simplified three-dimensional geometric model was directly imported into ICEM software to optimize the geometric model, initialize, divide, merge, and modify the block, set the size of the mesh and the type of mesh generation method to automatically generate surface and volume meshes, and finally perform mesh inspection to import meshes that do not meet the requirements after smoothing, thinning/roughening, merging, and automatic repairing (Wu et al., 2017). The sacral nerve was assumed to be a homogeneous cylindrical structure, which means the mechanical properties of the biomaterial used were assumed to be homogeneous and continuous in all directions. Each unit can maintain stability under stress. 1 mm thick hexahedral elements were used to discretize the geometric model (Figure 3A). The nerve root finite element model contains a total of 60 hexahedral elements and 102 nodes. The origin is located at the midpoint of the bottom surface of the model. The fixed grid intersection was selected as the subsequent intersection to be identified, and the method of measuring pixel points was used to measure the displacement of the selected point under different level of pressures. The material model selected the widely used viscoelastic model (*MATVISCOELASTIC) in LS-Dyna. Since only the sacral nerve is involved in the finite element calculation, the relevant data in the calculation comes from the elastic model. *MATVISCOELASTIC mainly includes five parameters—density, elastic volume model, short-term shear model, long-term shear modulus, and attenuation coefficient. The values of the initial material parameters refer to the muscle material in the commercial human body finite element model GHBMC.

Boundary condition: The *BOUNDARYSPCSET keyword was used to completely constrain the bottom node, and the *BOUNDARYPRESCRIBEDMOTIONSET keyword was used to give the top node a boundary condition that moves in the X direction. To improve the efficiency of the solution, the loading adopts a uniform acceleration form; the initial speed was 0 m/s, and the accelerated velocity was 10 m/s2, evenly distributed on each node of the nerve surface, and the system damping was set to ensure that the kinetic energy of the system was zero. Set a fixed rigid support surface on which the nerve is placed and define contact between them. Then set a moving rigid surface above the nerve to simulate compression of the nerve and define its contact with the nerve and the moving rigid surface moves down with an initial velocity of 0.01 m/s. After completing the definition of other simulation control cards, LS-Dyna finite element simulation solution software was used to calculate. The output parameters in the simulation process include nodal force and nodal displacement. After the simulation, the material parameters were adjusted according to the test data. After iterations, the material parameters obtained are shown in Table 3.

**TABLE 3:** Material parameters adjusted according to the test data.

RO	BULK (GPa)	GO	GI	BETA
1.0E-06 kg/mm³	0.0329	3.17E-04GPa	2.4E-04GPa	0.1

#### 2.7.4 Simulation of nerve root radial compression test

In the simulation of the nerve root radial compression test, the finite element model of the nerve root was consistent with the model used in the previous tensile test. However, appropriate changes to the boundary conditions of the simulation according to the test settings were required. The keyword *RIGIDWALLPLANAR was used to define the rigid surface of the lower support of the test piece, and the keyword *RIGIDWALLPLANARFORCESMOVING was used to define the upper compression plane (Figure 3B). The mass of downward pressure of the rigid surface was defined as 100 kg, which was pressed down at an initial velocity of 0.01 m/s and evenly distributed on each node of the nerve surface. After completing the definition of other simulation control cards, we used the LS-Dyna finite element simulation solution software to calculate. We set 4 analysis conditions: compression of 0 g, 10 g, 30 g, and 60 g. The output parameters in the simulation process included the displacement of the rigid surface and contact force.

#### 2.7.5 Validity verification of the finite element model

The biomechanical response of the nerve obtained by the simulation of the axial tension test showed that the corresponding tensile force was about 0.58 N when the displacement was 2.17 mm, which is consistent with the biomechanics of the nerve material in the test. Thus, we showed that finite element model is effective.

## 3 Results

### 3.1 Anatomy and electrophysiological study of lumbosacral nerve roots in SD rats

#### 3.1.1 Composition and measurement of lumbosacral nerve roots in SD rats

Anatomical studies have found that the lumbosacral plexus in rats has great variation, and the composition of femoral nerve, obturator nerve, sciatic nerve, pudendal nerve and coccygeal nerve trunks are inaccurate. Therefore, the premise of this study is to locate the nerve composition of the lumbosacral plexus and then to establish a stable model. The anatomical results of the composition of the femoral, obturator, sciatic, and pudendal nerves, and the tail nerve trunk are shown in Table 4. The data shows that L5 nerve constituted the sciatic nerve constantly, and the L6 nerve constituted the pudendal nerve constantly. The measured values of L4-S1 nerve root length, nerve root diameter, and intervertebral foramen diameter are shown in Table 5. The length of the L5 nerve root was 3.67 ± 0.15 mm, which is suitable for placing electrodes for subsequent electrophysiological tests.

**TABLE 4:** Neural composition of rats.

Nerve root	Nerve
L1+L2+L3+L4	femoral nerve + obturator nerve
L4+L5	sciatic nerve
L6+S1	pudendal nerve
S1+S2+S3+S4	coccygeal nerve trunk

**TABLE 5:** Measurement of distance between adjacent lumbosacral nerve foramen (mm).

	L4→L5	L5→L6	L6→S1	S1→S2
intervertebral foramen diameter	6.75 ± 0.07	5.65 ± 0.08	6.70 ± 0.12	5.86 ± 0.20
nerve root length	3.19 ± 0.12	3.67 ± 0.15	3.01 ± 0.22	4.5 ± 0.18
nerve root diameter	1.72 ± 0.11	1.80 ± 0.07	1.01 ± 0.09	0.85 ± 0.05

#### 3.1.2 Nerve conduction velocity

**FIGURE 4:**
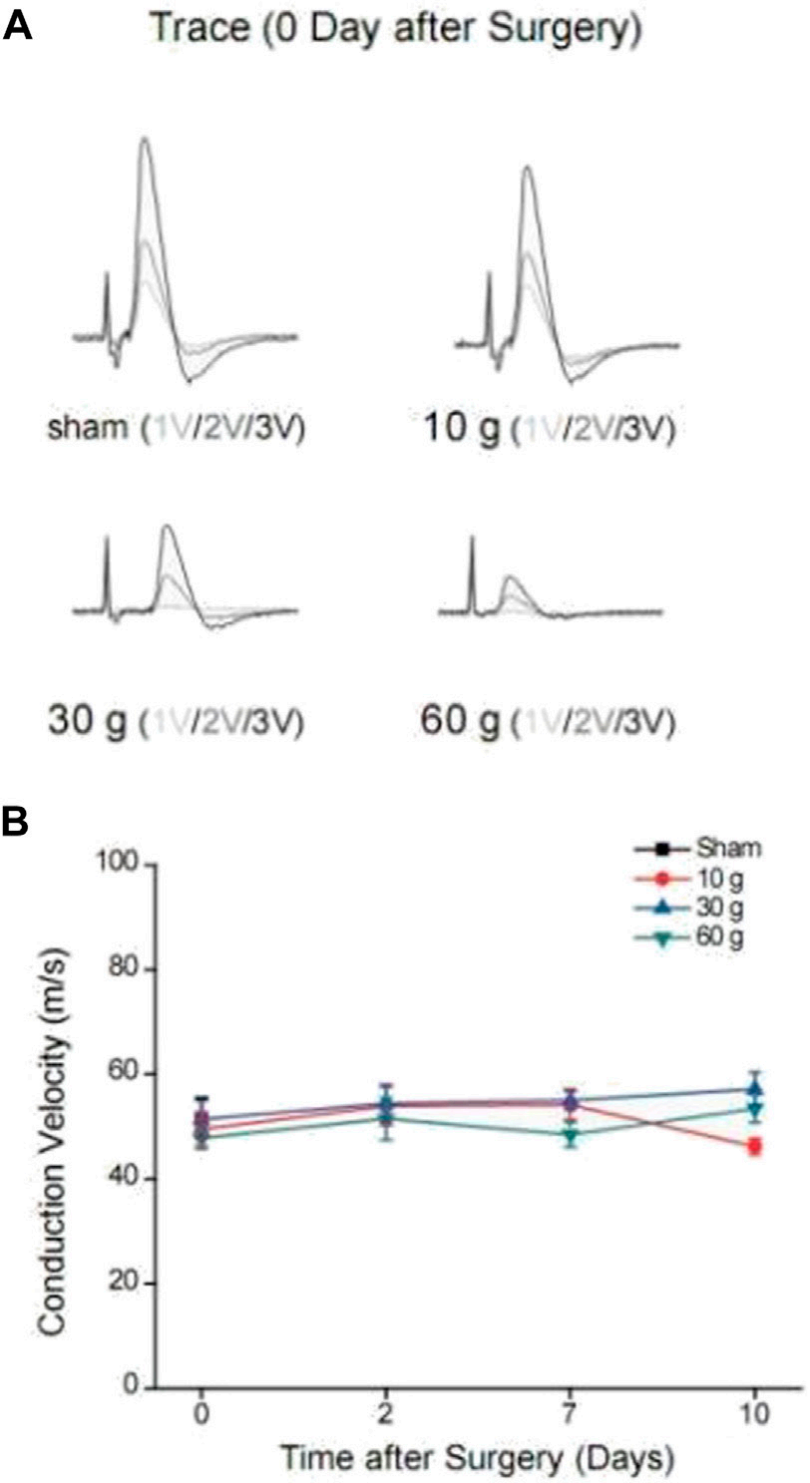
**(A)** Changes in waveforms of each group under different voltage stimulation 0d after compression, and CV change curve for each compression group and each time point, **(B)** CV curve of each compression group and each time point.

A stimulating electrode was placed on the sciatic nerve and two recording electrodes were placed on the L5 nerve root to record the changes in nerve conduction velocity after compression. The different time points in each group were compared longitudinally. At days 0, 2, 7, and 14 after compression, the conduction velocity did not change significantly. When each group was compared horizontally, the conduction velocity did not decrease significantly (Table 6). The electrophysiological waveforms of the 30 and 60 g compression groups showed that the first action potential time after the stimulus artifacts was significantly longer than that of the sham and 10 g groups. This change was not observed in the compression 10 g and sham groups (Figures 4A,B).

**TABLE 6:** Nerve conduction velocity (CV) of different compression levels at different time points.

Sham (d)		10 g/d	30 g/d	60 g/d
CV(m/s)		CV(m/s)	CV(m/s)	CV(m/s)
0	58.28 ± 6.71	61.43 ± 1.24	55.25 ± 3.54	59.42 ± 3.23
2	59.34 ± 7.63	59.87 ± 7.43	57.40 ± 6.43	60.03 ± 1.36
7	57.76 ± 13.55	57.07 ± 8.38	57.07 ± 1.16	56.99 ± 5.33
14	60.56 ± 4.18	56.45 ± 12.34	60.15 ± 2.43	58.86 ± 7.12

#### 3.1.3 Area of curve under compound action potential

**FIGURE 5:**
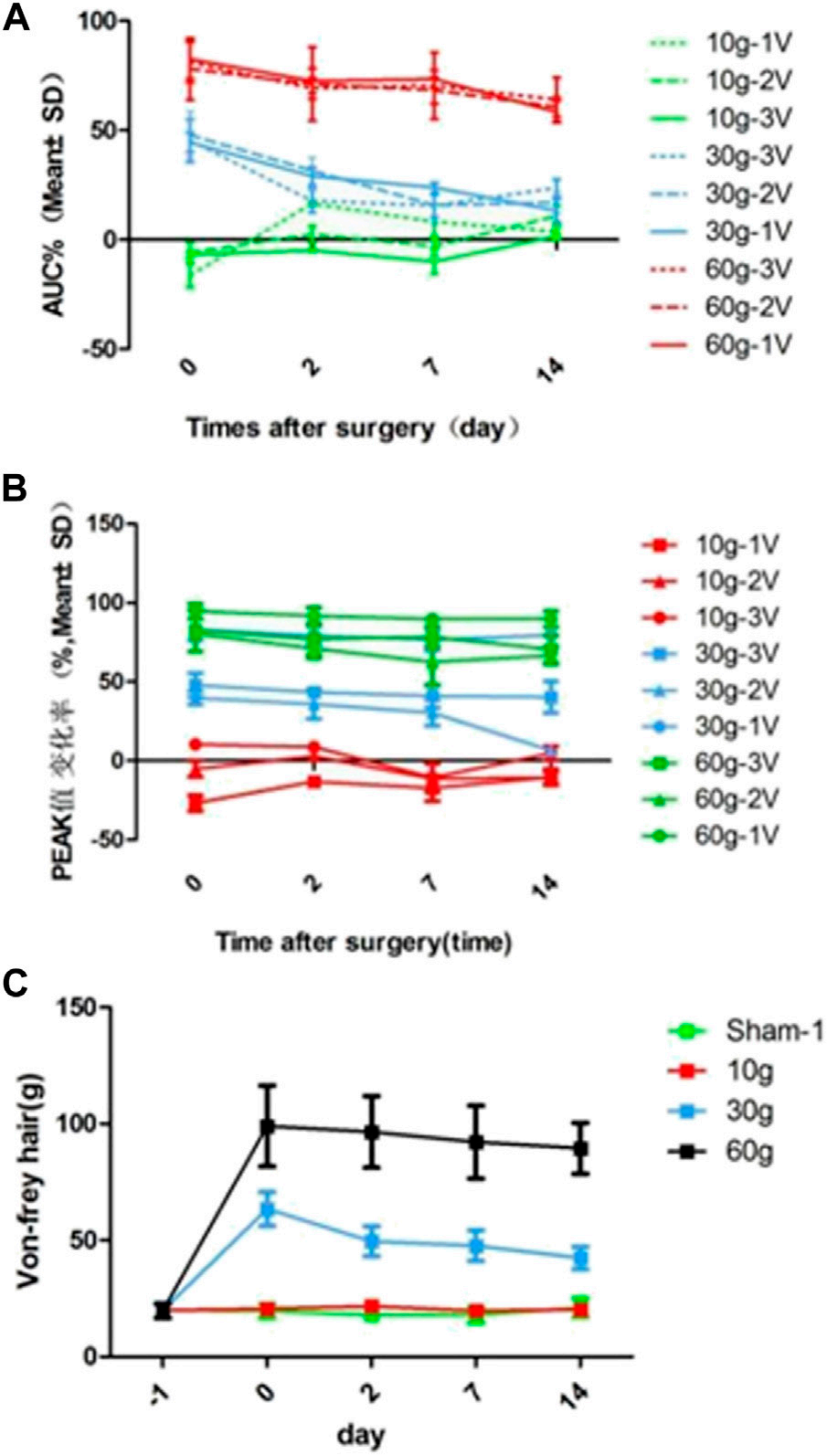
**(A)** AUC reduction rate curve for each compression group and each time point, **(B)** Curve of reduction rate of PEAK value for each compression group and each time point, **(C)** von Frey hair force value change curve for each compression group and each time point.

The area under the curve of the compound action potential (AUC) reflects the nerve activation after electrical stimulation. On day 0 after 10 g compression, 3 V, 2 V, and 1 V were used for immediate stimulation, and the area of the curve under the compound action potential (AUC) increased by 6.64 ± 1.34%, 5.83 ± 4.46%, and 16.37 ± 5.52% respectively. On day 0 after 30 g compression, AUC of the compound action potential was immediately stimulated with 3 V, 2 V, and 1 V, and AUC reduced 5.27 ± 9.85%, 47.84 ± 10.63%, and 44.48 ± 4.46% respectively. On day 0 after 60 g compression, under 3 V, 2 V, and 1 V stimulation, the AUC of the compound action potential reduced 81.12 ± 9.25%, 78.09 ± 14.12%, 82.52 ± 9.01% respectively. After 10 g compression, using 1 V voltage to stimulate the nerve at 0–14 d, AUC decreased by 16.49 ± 0.97% at 2d, 8.23 ± 7.87% at 7d, and 3.64 ± 3.55% at 14d. The 2 and 3 V stimulation also showed an increasing trend at 2d and 7d, and decreased on 14d, by 10.8 ± 4.73% and 1.701 ± 0.96% respectively. 30 and 60 g compression showed that AUC of the 0d composite action potential in groups of 3 V, 2 V, and 1 V were reduced by 45.27 ± 9.85%, 47.84 ± 10.63%, and 44.48 ± 4.46%, and 81.12 ± 9.25%, 78.09 ± 14.12%, and 82.52 ± 9.01% respectively. As time increased, the percentage of AUC of the compound action potential of decrease gradually decreased (Table 7 and Figure 5A).

**TABLE 7:** The percentage of reduction in AUC value at different time points for different levels of compression.

Group/day (g)		3V AUC%	2V AUC%	1V AUC%
10	0 day	−6.64 ± 1.34	−5.83 ± 4.46	−16.37 ± 5.52
	2 day	−4.81 ± 0.61	2.47 ± 3.88	16.49 ± 0.97
	7 day	−9.96 ± 5.74	−2.95 ± 4.49	8.23 ± 7.87
	14 day	1.701 ± 0.96	10.8 ± 4.73	3.64 ± 3.55
30	0 day	45.27 ± 9.85	47.84 ± 10.63	44.48 ± 4.46
	2 day	17.78 ± 5.40	31.51 ± 5.93	29.04 ± 4.11
	7 day	15.54 ± 5.15	15.77 ± 8.72	23.91 ± 2.19
	14 day	23.71 ± 3.97	16.92 ± 4.87	13.35 ± 5.25
60	0 day	81.12 ± 9.25	78.09 ± 14.12	82.52 ± 9.01
	2 day	69.13 ± 4.83	71.15 ± 16.85	72.62 ± 5.67
	7 day	70.45 ± 15.28	68.26 ± 6.04	73.39 ± 4.08
	14 day	64.04 ± 9.96	60.68 ± 4.41	58.46 ± 4.96

#### 3.1.4 Peak value of nerve compound action potential

The peak value reflects the amplitude change of the maximum action potential after nerve activation. With stimulation immediately after 10g compression, peak values at 1 and 2 V increased by 26.83 ± 4.9% and 5.02 ± 5.13% respectively, and 3 V decreased by 10.42 ± 3.34%. With 3 V, 2 V, 1 V stimulation immediately after 30 g compression, the peak value decreased by 47.93 ± 7.57%, 39.82 ± 4.12%, and 83.25 ± 6.88% respectively. The peak values were reduced by 82.49 ± 13.23%, 80.40 ± 2.01%, and 94.72 ± 4.77% respectively with 3 V, 2 V, and 1 V stimulation immediately after 60 g compression on the 0th day. There were significant differences among the three groups (*p* < 0.05). In the 10 g group, the peak value of 0d-14d after 1 V stimulation increased by 26.83 ± 4.9%, 13.03 ± 3.7%, 17.3 ± 8.10%, and 10.24 ± 5% respectively, and the peak value of 0d-14d with 2 V stimulation increased 5.02 ± 5.13%, 2.92 ± 2.49% (Decrease, 2d), 11.21 ± 2.50%, and 10.75 ± 4% after compression. In the 3 V stimulation group, peak value decreased by 10.42 ± 3.34%, 8.83 ± 1.70%, 10.66 ± 8.88% (increase, 7d), and 4.72 ± 4.49% respectively. There was a significant difference between 2d, 7d, 14d, and 0d in the 1V group (*p* < 0.05), and there was no significant difference between the other groups (*p* > 0.05). There was no significant difference between the 30 and 60 g groups at each time point (*p* > 0.05; Table 8, Figure 5B).

**TABLE 8:** Percentage of reduction of PEAK value at different time points for different compression levels.

Group/day (g)		3V PEAK%	2V PEAK%	1V PEAK%
10	0 day	10.42 ± 3.34	−5.02 ± 5.13	−26.83 ± 4.9
	2 days	8.83 ± 1.70	2.92 ± 2.49	−13.03 ± 3.7
	7 days	−10.66 ± 8.88	−11.21 ± 2.50	−17.3 ± 8.10
	14 days	4.72 ± 4.49	−10.75 ± 4	−10.24 ± 5
30	0 days	47.93 ± 7.57	39.82 ± 4.12	83.25 ± 6.88
	2 days	43.49 ± 2.34	35.95 ± 9.34	79.32 ± 5.84
	7 days	41.01 ± 7.37	30.37 ± 8.1	76.66 ± 6.67
	14 days	40.26 ± 9.96	36.55 ± 1.29	79.51 ± 0.02
60	0 days	82.49 ± 13.23	80.40 ± 2.01	94.72 ± 4.77
	2 days	77.12 ± 9.84	71.15 ± 6.29	91.68 ± 5.39
	7 days	78.49 ± 6.47	62.58 ± 14.94	89.76 ± 1.96
	14 days	70.52 ± 8.68	66.62 ± 5.46	89.75 ± 5.01

#### 3.1.5 Von-frey hair force

The L5 nerve sensory innervation area of the SD rat foot was located and von Frey hair was used to stimulate the area to observe nerve discharge. There was no significant difference between the groups before compression. There was also no significant difference at each time point in the 10 g compression group. Compared with the sham and 10 g groups, the strength was significantly increased after 30 and 60 g compression (*p* < 0.05). After 30 g compression, the strength at 0 d, 2d, 7d, and 14d was 63.45 ± 7.25 g, 49.56 ± 6.41 g, 47.71 ± 6.58 g, and 42.38 ± 4.84 g respectively. When strength at 0d, 2d, 7d and 14d were compared, a significant decrease was observed in the 2d, 7d, and 14d (*p* < 0.05). There was no significant difference between the 2d, 7d, and 14d groups (*p* > 0.05). There was no significant difference between each time point after 60 g compression (Table 9; Figure 5C).

**TABLE 9:** Von frey hair force values of different compression levels at different time points.

Day	Sham-1	10g	30g	60g
before	20.08 ± 1.98	19.96 ± 1.73	19.98 ± 3.08	19.63 ± 3.03
0d	19.68 ± 2.95	20.53 ± 2.66	63.45 ± 7.25	99.06 ± 17.32
2d	17.9 ± 1.95	21.68 ± 2.41	49.56 ± 6.41	96.53 ± 15.39
7d	17.91 ± 3.36	19.65 ± 2.21	47.71 ± 6.58	92.18 ± 15.58
14d	21.03 ± 4.03	20.31 ± 2.53	42.38 ± 4.84	89.55 ± 10.94

### 3.2 Biomechanical study of the nerve root, lumbosacral trunk and sacrum

#### 3.2.1 Biomechanical test of the rat nerve root and lumbosacral trunk

**FIGURE 6:**
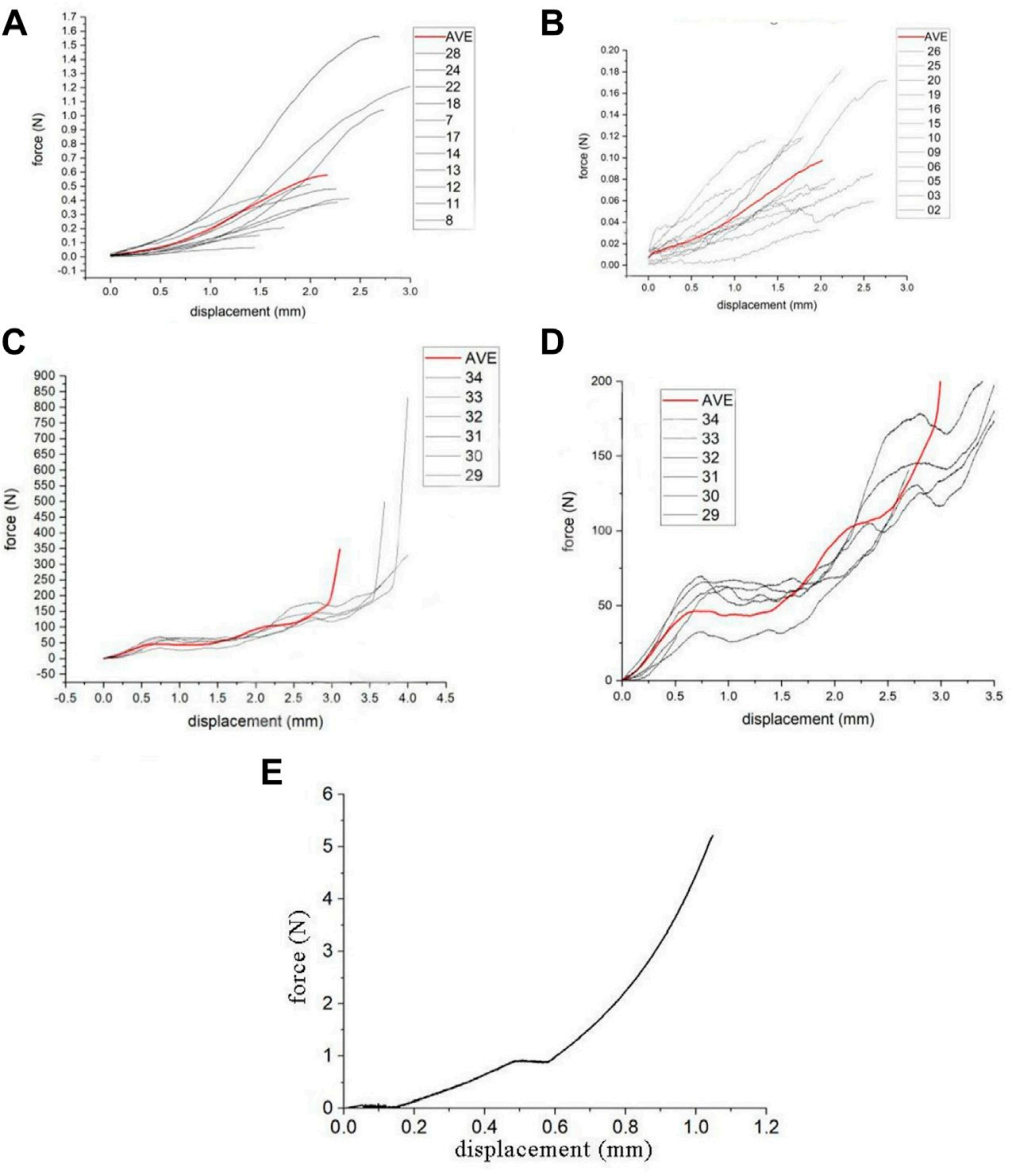
**(A)** Curve in lumber trunk test, **(B)** Curve in nerve root test, **(C)** Lumbar vertebrae compression test curve, **(D)** Partial enlarged view of lumbar vertebrae compression test curve, **(E)** The compression force-displacement curve obtained using the radial compression test simulation.

As shown in the tensile force-displacement test curve of the lumbosacral trunk (Figure 6A), the lumbosacral trunk nerve test showed that the maximum bearable load could reach 1.56 N. The average maximum load was 0.58N. The tension-displacement test curve of the nerve root (Figure 6B) showed that the maximum bearable load for the nerve root was 0.19 N, and the average maximum load was 0.097N. INSTRON 5985 biomaterial testing machine was used to obtain the material parameters of the rat nerve roots and lumbosacral stem. The data showed that the load on the nerve root is lower than that on the lumbosacral trunk, indicating that the diameter of the nerve is the main factor determining its material properties. This shows that the nerve root is more vulnerable to damage than the peripheral nerve. According to the classical mechanics formula of the material tensile test, the engineering stress-strain curve of each sample can be obtained, which represent the biomechanical properties of neural materials that do not depend on geometry (Figures 2A,B). Among them, the red curve represents the average engineering stress-strain curve. The average ultimate stress of the lumbosacral trunk (Figure 3A) was 0.530 MPa, and the average ultimate strain was 48.9%. As shown in Figure 3B, the average ultimate stress of the nerve root was 0.377MPa, and the average ultimate strain was 28.9%.

#### 3.2.2 Biomechanical testing of the rat sacrum

The rat sacrum was subjected to a uniform compression test using INSTRON 5985 at a compression speed of 0.02 mm/s. Before the experiment, a 0.2 N preload was applied to the sample. The compression force-displacement test curve of the sacrum and its partial enlarged view are shown in Figures 6C,D The sacrum compression force-displacement test curve showed that the sacrum would undergo a two-stage rupture process during the transverse compression process. The first stage of collapse corresponds to the collapse of the sacral foramen, and the pressure is 32–70 N, with an average of 47 N. The second stage of collapse corresponds to the collapse of the vertebral body, with an average force of about 110 N. The sacrum Denis Zone Ⅱ and Ⅲ areas (sacral foramen) are the weakest positions in the entire sacrum, and will fracture first under external force.

### 3.3 Finite element analysis of sacral nerve constriction injury caused by sacral fracture

The image was used to obtain the geometric parameters before the nerve tensile test and the engineering stress-strain curve of each sample. The maximum stress that the lumbosacral trunk can withstand in tensile load is 0.530 MPa, while that for the nerve root is 0.377 MPa. The biomechanical response of the nerve obtained by the simulation of the axial tensile test showed that the corresponding tensile force is about 0.58 N when the displacement is 2.17 mm, which is consistent with the biomechanics of the nerve material in the test. This material parameter could be used to simulate the biomechanical response of a nerve under compression. The compression force-displacement curve obtained by the radial compression test simulation is shown in Figure 6E. The force of the nerve is about 0.36 N at 25% compression, 0.91 N at 50%, and 2.94 N at 75%. The maximum force when the compression reaches 90% is approximately 5.2 N. According to the results of the lateral compression test of the sacrum, the crushing force of the vertebral foramen is about 32–70 N, with an average of 47 N. Thus, the maximum load that the nerve can withstand is only 5.2 N, which is much lower than the lateral crushing force of the sacral foramen. Thus, when the sacrum is crushed, the nerves will inevitably be damaged.

## 4 Discussion

### 4.1 Anatomical characteristics of lumbosacral nerve roots and lumbosacral nerve root constrictive injury model in SD rats

The SD rat has 6 segments of lumbar vertebrae and 4 segments of sacral vertebrae. The spinal cord of SD rats is shorter than the spinal canal, which is similar to that of humans (Zhou et al., 2018). After the nerve roots are emitted, they travel a certain distance in the spinal canal before they can pass through the corresponding intervertebral foramen and four pairs of sacral nerves (S1-S4) penetrate the sacral foramen. L1-L4 constitute the femoral and obturator nerves; the sciatic nerve is constituted by L4-6, the pudendal nerve is composed of L6-S1, and the caudal nerve trunk is comprised of S1-4.

In humans, L5 nerve root runs in the nerve groove on the surface of the sacral promontory, after crossing the sacral promontory, it meets with L4 nerve root to form the lumbosacral trunk. The S1-4 sacral nerves run in the nerve groove on the lateral side of the sacrum after they emerge from the sacral foramen. The S1 nerve root joins with the lumbosacral trunk in front of the sacroiliac joint, and then merges with S2, 3, and 4 in front of the piriformis muscle, and finally forms two terminal branches: the sciatic nerve and the pudendal nerve. Nerve damage caused by sacral fractures mainly includes lumbosacral stem (L4, 5) and sacral nerve root damage in humans. In unstable pelvic fractures, especially Tile C type, traction injuries to the lumbosacral trunk and nerve roots are common. In sacral Denis Zone Ⅱ fractures, the sacral nerve root constrictive injury caused by the deformation of the sacral foramen is the main cause of injury. Sciatic nerve (S1) injury is the most common in such injury, and patients suffering from this type of injury typically show paresthesia and hypokinesia (Zochodne, 2012), which is easily observed and evaluated in the clinic. Therefore, we take the S1 nerve injury caused by sacral fracture as the research object, and then we can deduce the S2, 3, 4 injuries after the results are obtained.

In rats, the L5 nerve root constitutes the sciatic nerve consistently. In order to obtain more accurate experimental results, we placed the stimulating electrode on the sciatic nerve and the recording electrode on the L5 nerve root, through which we simulate human S1 nerve root injury. The length of the L5 nerve root is 3.67 ± 0.15 mm and the diameter is 1.80 ± 0.07 mm. There is enough space to place vascular clips and electrodes. Furthermore, we can effectively reduce the artificial damage caused by the experimental operation.

### 4.2 Evaluation of nerve function in rat lumbosacral nerve root constriction injury

Methods such as postoperative evaluation of symptoms, neurological index, and electrophysiology of animal models are generally used to evaluate nerve root injury, (Gordon and Borschel, 2017).

With the continuous progress of electrophysiological technology research, electrophysiology not only provides effective assessments of the scope and extent of peripheral nerve damage, but can also show the degree of peripheral nerve repair (Sun et al., 2014).

In this study, when we administered a standardized crush injury to the lumbar 5 nerve roots of SD rats, axon continuity was lost, which resulted in Waller’s degeneration of distal injury. Early intervention inhibited the high-frequency stimulation of afferent nerves and decreased the transmission of pain to the higher central (Zhou et al., 2018).

Bendszus et al. (2004) studied the electrophysiology of rat sciatic nerve during controlled degeneration and regeneration after ligation. After injury, the composite muscle action potential (CMAP) and electromyography measurements dropped sharply, and gradually increased within 7 weeks after injury, coinciding with histological evidence of regeneration. Thus, neuroelectrophysiology could provide an accurate assessment of nerve damage.

In this study, electrophysiological detection methods were used to evaluate the functional changes in nerve roots after compression, and to determine the electrophysiological characteristics of nerve roots that change over time under different degrees of compression.

### 4.3 Changes in nerve conduction velocity

Nerve conduction velocity (CV) refers to the distance that a nerve impulse passes through nerve tissue in a unit of time. By measuring the time difference and distance between the occurrence of compound action potentials on two recording electrodes, we can calculate the nerve conduction velocity (Lin et al., 2018). Nerve conduction velocity testing is often used to check the functional status of neurons, and diagnose whether diseases such as amyotrophic lateral sclerosis and myasthenia gravis come from the nervous or skeletal muscle system. Therefore, nerve conduction velocity (CV) measurement is a good indicator of nerve function. Because it is relatively non-invasive and reusable, it is often used to evaluate nerve function and diagnose nerve and muscle diseases and sensory disorders (Suzuki et al., 2017; Kizilay et al., 2019).

We measured the nerve conduction velocity (CV) of the lumbar 5 nerve root of SD rats, and found no significant difference in conduction velocity from 0d to 14d between the 10 g, 30 g, and 60 g groups. In the 30 and 60 g compression groups, the electrophysiological waveforms showed that the first action potential time after stimulation artifacts was significantly longer than that in the sham and 10 g groups. The results of this experiment showed that the degree of sciatic nerve damage increased with the continuous increase of compression force. However, conduction function could still be preserved after nerve root compression injury, and the nerve root may gradually recover its function.

### 4.4 Changes in the area of the curve under compound action potentials

After 30 and 60 g compression, AUC of the 0d composite action potential decreased and was positively correlated with the degree of compression. The percentage of AUC of the compound action potential decreased gradually with time. The highest decrease in AUC under the 30 and 60 g compression compound action potentials was observed at 0d, and improved with time. Therefore, compression over 30 g could significantly affect nerve function. In the acute period of nerve injury, the nerve fibers farther away from the nerve injury point do not undergo avascular necrosis, and the electrophysiological action potential capacity can remain for several days. When the nerve root was under 10 g compression, AUC under the compound action potential increased, indicating that low degrees of compression could cause abnormal nerve discharge. When 10 g compression was measured on days 2, 7, and 14, AUC under the 1 V stimulation compound action potential decreased, and the decrease was negatively correlated with time. Under 2 and 3 V stimulation, the AUC showed an increasing trend at 2d and 7d, and decreased at 14d. Thus, the abnormal discharge of the nerve is reduced after 2d, and 1V stimulation could not cause the abnormal discharge, which could be activated by stronger stimulation (2 V, 3 V). By the 14th day, AUC under the compound action potential stimulated by 1–3 V returned to normal.

### 4.5 Changes in the peak value of nerve compound action potential

The peak value of nerve compound action potential (peak value) reflects the amplitude change of the maximum action potential after nerve activation. The results of this experiment showed that after 10 g compression with 3 V, 2 V, or 1 V stimulation, peak value increased by 26.83 ± 4.9%, 5.02 ± 5.13% at 1 and 2 V respectively, and decreased by 10.42 ± 3.34% at 3 V. Similar to the results of AUC, 10 g compression caused nerve irritability. The peak value of the 3 V nerve compound action potential decreased, but AUC increased, indicating that more abnormal nerve discharges were activated at 3 V. Peak value showed no obvious change with time. The results of AUC after 30 and 60 g compression were similar, Peak value decreased and was positively correlated with the degree of compression, as previously reported (Hemmi et al., 2018).

### 4.6 Changes in von frey hair force

We used the von Frey hair method to assess changes in neurosensory function (Wu et al., 2017). 10 g clamping of nerve roots did not cause significant changes in sensory function. Von Frey hair force increased significantly in the 30 and 60 g compression groups compared with that in the sham and 10 g groups. It was 63.45 ± 7.25 at 0d in the 30 g compression group, and the sensory function recovered with time. At 2d, 7d, and 14d von Frey hair force significantly decreased (*p* < 0.05). However, there was no significant difference between the 2d, 7d, and 14d groups (*p* > 0.05). There was no significant difference after 60g compression at each time point. Thus, compression with a weight <10 g causes no obvious damage to the sensory function of the sciatic nerve, while 30 g compression of the sciatic nerve could increase sensory function damage with prolonged compression time. 60 g compression caused severe damage to the sensory function of the sciatic nerve in a short time.

### 4.7 Biomechanical features of sacral nerve and sacrum injury

Understanding the biomechanical properties of nerve is essential to study the mechanism of injury. Compared with traction injuries, nerve constriction injuries typically show better recovery. Dahlin et al. reported that in addition to the nerve damage caused by the pressure itself, there are other effects that cause nerve damage and edema (Dahlin and McLean, 1986). Although sacral nerve damage caused by sacral fractures have been widely recognized, there have been very few studies on the specific mechanisms and related mechanics underlying sacral nerve damage. A biomechanical model of sacral nerve injury is therefore helpful to study the biomechanical changes and identify the causal factors.

Isolated tissues can be transformed from a static state to a moving state by pretreatment, so that the mechanical properties of the test tissue have a certain degree of stability and repeatability (Fung and Skalak, 1981). The INSTRON 5985 testing machine with the tensile speed set to a stretching velocity of 0.01 mm/s is used to perform a uniform tension test, which is a replacement for direct compression. The sacrum, as a constant-stress structure, has a parabolic distribution of stress and shows characteristics of stress concentration. The stress concentration in the sacral foramen is the most obvious. When the sacrum is subjected to trauma, the stress of the sacrum foramen can reach about 3 times that of the sacrum (Wheeldon et al., 2006). Our results are similar to those in the literature: when the sacral foramen breaks, all the forces are 47 N on average, and the force on the sacral foramen is about 110 N. The stress concentrating in the sacral foramen may be the reason for the first involvement of the sacral foramen after trauma. In our sacral biomechanics experiments, the sacrum of rats were subjected to a compression test. During the compression and break process, the sacral foramen was crushed first, followed by the sacral part. When the sacrum fractures, the pressure-stress curve has a certain linear relationship. We found that sacrum fracture occurred in two stages when transverse compression occurs. The existence of uniform pressure between the two stages could lead to the phenomenon that the displacement changes. It is believed that this is related to the involvement of the sacral foramen.

The sacral nerve is a viscoelastic tissue, and its biomechanical properties are related to the stress-strain relationship and stress relaxation. There is no correlation between nerve function assessment and histomorphometric analysis (Munro et al., 1998). Conversely, the severity of nerve damage may be affected by two main factors: pressure and squeeze time (Dahlin et al., 1986). The biomechanical changes in nerves before and after injury are mainly affected by the epineurium and perineurium. When the diameter of the nerve is larger, the nerve also has greater stiffness (Ngeow et al., 2011). In an experiment to study the mechanical properties of the ultrastructure of the rat sciatic nerve, the thickness of the nerve membrane was assumed to be 3% of the nerve diameter. The larger the nerve diameter, the stronger the resistance to compressive load (Chang et al., 2015). Previous studies of nerves have presented questions on how damaged nerves respond to increased load, and whether it has an impact on the definition of the degree of damage, that is, whether there is a level that defines the severity of nerve damage (Mahan et al., 2019). We believe that functional recovery and the possibility of damage after nerve injury are related to the characteristics of nerve materials, which are related to the diameter of the compressed nerve. Due to the different mechanisms of nerve damage caused by nerve traction and compression, in order to find the correlation between nerve damage and the characteristics of nerve materials, we used uniform traction speed, and unified variables such as the size of the trauma and the action time of the nerve to reduce interference with the material characteristics of the nerve itself, and thereby reduce the impact of the damage mechanism on the experimental results. Since nerves are a type of viscoelastic material, they do not tend to concentrate like rigid materials, making the tensile force-displacement test relatively scattered. Nerve roots and peripheral nerves have different mechanical properties. Nerve roots are more likely to be injured than peripheral nerves (Ngeow et al., 2011). In our previous research, we found that the diameter of the nerve root (average 0.6 mm) is smaller than that of the lumbosacral trunk (average 1.1 mm), which is calculated based on the measurement results of the lumbosacral trunk and nerve root load-displacement in the biomechanical experiments. Comparing the maximum stress, maximum stress strain, elastic variables, and material parameters of the nerve root and lumbosacral trunk, showed that the capacity to bear load of the lumbosacral trunk is much higher than that of the nerve root, indicating that the nerve root is more susceptible to injury than the peripheral nerve. The biomechanical properties of the injured nerve are therefore likely determined by the diameter of the damaged nerve.

### 4.8 Finite element analysis of sacral nerve injury

We established a three-dimensional living body structure model, and assigned the structure characteristics of neurobiomaterials to the model to obtain a virtual experimental specimen. The model was loaded with different experimental conditions to simulate the relationship between sacral fracture compression and sacral nerve injury. The establishment of a finite element model of sacral nerve injury is beneficial to the study of the degree of nerve injury and the effectiveness of nerve decompression, and could guides clinical treatment (Ma et al., 2011). However, the rat sacral nerve is a soft tissue, and its CT images are difficult to obtain. To reconstruct the finite element model of the rat sciatic nerve based on MRI images, the surrounding muscle, bone tissue, and other structures must be imaged, and the operation is complicated. Because the bone tissue is not clear in MRI images, it is difficult to establish an accurate model. Therefore, we used reverse modeling technology.

In this study, the material parameters of the nerve were determined by simulating the tensile test, and were used to simulate the load that the nerve can bear when it is compressed. Although the real nerve bundle is composed of more fibers of different diameters, it is necessary to use simplified geometry to evaluate the electromechanical equivalence in the 3D finite element model and limit the computational cost (Walia et al., 2017). The mechanical properties of the biomaterials involved in this experiment were assumed to be homogeneous and continuous. Each element of the model has sufficient stability when subjected to force, regardless of the deformation of the material. In addition, a balance between bio fidelity and computational efficiency must be found. Since nerves are soft tissues, it is difficult to measure their geometric shape. Finite elements are used in soft tissues such as blood vessels and tendons. In the rat anatomy experiment, the length of the L5 nerve root is 3.67 ± 0.15 mm and the diameter is 1.72 ± 0.11 mm. Therefore, it is also likely feasible to simplify the sacral nerve to a cylindrical structure with a diameter of 1.7 mm and a height of 3 mm. Many biological tissues are anisotropic, heterogeneous. Due to the small size and softness of neural tissue, it is difficult to perform precise mechanical tests to obtain accurate material constitutive parameters, so many neurobiomechanical studies still assume it as an isotropic material. In the ophthalmology study conducted by Karimi et al. (2021), the lamina cribrosa, as a small structure that is difficult to image *in vitro* and *in vivo*, was assumed as a homogenized continuum material in the finite element modeling; The optic nerve is often studied with finite element analysis under the assumption that it is isotropic (Wang et al., 2016; Shin et al., 2017; Safa et al., 2021). In animal modeling, some scholars have assumed that the muscles, glands, and connective tissue around the cat’s urethra are isotropic (Woock et al., 2010). In the study of the central nervous system, there are also examples of experimental design by Maikos et al. (2008) assuming that the rat spinal cord is isotropic and homogenous. We therefore assume that the sacral nerve is a homogeneous cylinder, and combined the classical mechanics principles in the material experiment to obtain the stress-strain curve of each sample, and then inversely estimate the neural material parameters. In study, the mass of the pressed rigid surface was defined as 100 kg, and pressed at an initial speed of 0.01 m/s to obtain the relevant values of each node in the model. According to the nerve biomechanical response obtained from simulation of the axial tensile test, the corresponding tensile force was about 0.58 N when the displacement was 2.17 mm, which is consistent with the biomechanical response of the nerve material (the average maximum load that the lumbosacral trunk can bear 0.58N) in the biomechanical test. This material parameter could therefore be used to simulate the biomechanical response of a nerve under compression and to verify the validity of the three-dimensional finite element model of the sacral nerve. It provides a scientific research method for further studying the relationship between the degree of sacral fracture compression and the degree of sacral nerve damage caused by the fracture.

Clinically, for fractures caused by violent lateral compression of the sacrum in human patients, the degree of compression of the sacrum can be evaluated by CT scan. However, the degree of damage of the nerve cannot be specifically evaluated. Through finite element analysis, we can simulate the compression process of the sacral foramen. According to the simulation of the radial compression test, the force of the nerve is 0.36 N when the sacral foramen is compressed at 25%, about 0.91 N at 50%, and about 2.94 N at 75%. The maximum force at 90% is about 5.2 N. Through the study of sacral biomechanics, we found that the compressive force to cause the rupture of the vertebral foramen is 32–70 N, with an average of 47 N. Thus, the maximum load that the sacral nerve can bear is much lower than the lateral crushing force experienced when the sacral foramen is crushed. Therefore, under severe sacral foramen pressure, sacral nerve damage is inevitable. In order to study the relationship between the degree of fracture and nerve damage, we combined this data with that from previous neuroelectrophysiological experiments and found that when the compression force is 30 g and the compression degree of the sacral foramen is about 22.94%, the sacral nerve begins to show functional damage. When the compression is 60 g, the compression degree of the sacral foramen is about 33.16%, and the function of the sacral nerve will be irreversibly damaged. Through the combination of early neuroelectrophysiology, the degree of nerve damage can be judged according to the degree of compression of the sacral foramen, and can provide guidance for effective surgical decompression. Although there are certain differences between humans and rats, fresh human sacral nerve specimens cannot be obtained, making it difficult obtain this data in humans, the relevant data of rats are therefore valuable.

The treatment and clinical manifestations of sacral nerve injury have been extensively studied and researched by scholars in both humans and animals recent years. Biological research on sacral nerve injury mainly focuses on the defecation and urination behavior of animals. This is because cauda equina syndrome, one of the complications of sacral nerve injury, will greatly affect the quality of life, even result in anxiety of patients (Hicken et al., 2001). Wang et al. applied a minimally invasive surgical approach to insert a neuromodulation device through the sacral foramen to stimulate the nerve roots of the sacral segment to restore defecation after spinal cord injury (Wang et al., 2021). Zhu et al. also used sacral nerve electrical stimulation to improve defecation function and accelerate the recovery of colonic transmission functions in rat model of acute spinal cord injury (Zhu et al., 2020). Nerve transplantation has also been attempted to repair bladder dysfunction caused by related sacral nerve injuries (Barbe et al., 2021). However, these studies are all aimed at the recovery and regeneration of nerve function after sacral nerve injury rather than discussing specific treatment and prognosis prediction according to the degree of sacral foramen compression and sacral nerve injury from a clinical point of view. On the contrary, our research focuses on the recovery of nerve function after sacral fracture in rats subjected to different pressures, which has potential guiding significance for incision decompression in patients with sacral fracture in clinical work.

The disadvantage of this experiment is that the selected neural material is assumed to be homogeneous, the nerve actually is not homogeneous, but part of heterogeneity, which is an inherent defect of this method. In addition, the realistic nerve bundles are composed of more fibers of different calibers, but since the target of the study is a single nerve, this interference is less affected in our experiments.

## 5 Conclusion

The damage of sacral nerve after sacral foramen collapse is the main cause of nerve insufficiency and late disability in patients. Research on sacral nerve injury corresponding to different degrees of sacral foramen compression has the potential to predict neurological function and determine the indication for surgical decompression.

In this study, we showed that the neurological loss was positively correlated with the degree of compression. After early decompression, nerve root function recovery is possible after moderate compression; however, in severe compression group, the nerve function would not recover even after early decompression. Then, we successfully simulated nerve compression during sacral fracture with finite element analysis. Combining the results of the radial compression test simulation and the sacral compression test, it can be deduced that the damage of the sacral nerve is inevitable when the sacral foramen is deformed by pressure. Furthermore, we obtain the relationship between sacral foramen deformation and nerve function loss in rat model. Biomechanical study showed that the diameter of the nerve is the main factor determining its material properties. So, in the same degree of constriction, the nerve of human may bear more force compare than rat.

## Data Availability

The original contributions presented in the study are included in the article/supplementary material, further inquiries can be directed to the corresponding author.
